# STAT1 signaling protects self-reactive T cells from control by innate cells during neuroinflammation

**DOI:** 10.1172/jci.insight.148222

**Published:** 2022-06-22

**Authors:** Carlos A. Arbelaez, Pushpalatha Palle, Jonathan Charaix, Estelle Bettelli

**Affiliations:** 1 Center for Fundamental Immunology, Benaroya Research Institute at Virginia Mason, Seattle, Washington, USA.; 2 Department of Immunology, University of Washington School of Medicine, Seattle, Washington, USA.

**Keywords:** Autoimmunity, Cytokines, Multiple sclerosis, T cells

## Abstract

The transcription factor STAT1 plays a critical role in modulating the differentiation of CD4^+^ T cells producing IL-17 and GM-CSF, which promote the development of experimental autoimmune encephalomyelitis (EAE), an animal model of multiple sclerosis (MS). The protective role of STAT1 in MS and EAE has been largely attributed to its ability to limit pathogenic Th cells and promote Tregs. Using mice with selective deletion of STAT1 in T cells (STAT1^CD4-Cre^), we identified a potentially novel mechanism by which STAT1 regulates neuroinflammation independently of Foxp3^+^ Tregs. STAT1-deficient effector T cells became the target of NK cell–mediated killing, limiting their capacity to induce EAE. STAT1-deficient T cells promoted their own killing by producing more IL-2 that, in return, activated NK cells. Elimination of NK cells restored EAE susceptibility in STAT1^CD4-Cre^ mice. Therefore, our study suggests that the STAT1 pathway can be manipulated to limit autoreactive T cells during autoimmunity directed against the CNS.

## Graphical abstract

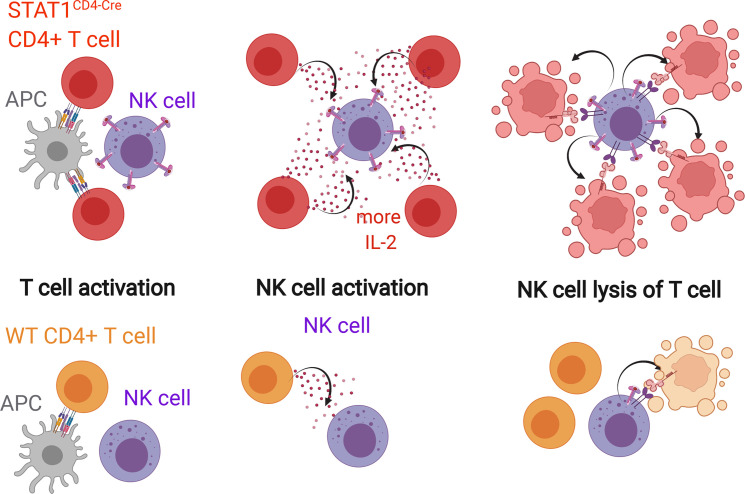

## Introduction

Multiple sclerosis (MS) is a disease characterized by inflammation, demyelination, and neurodegeneration of the CNS (1). It is believed to be an autoimmune disease initiated by CD4^+^ Th cells (2). Experimental autoimmune encephalomyelitis (EAE), which serves as an animal model of MS, is also induced by the activation of myelin reactive CD4^+^ T cells and their subsequent migration in the CNS, where they mediate inflammatory responses, resulting in demyelination and neurodegeneration. CD4^+^ T cells with the potential to induce EAE include subsets secreting IL-17, and GM-CSF with concomitant expression of IFN-γ (3).

Effector CD4^+^ T cell subsets have been defined based on their secretion of cytokines, as well as based on transcription factors and cytokines required for their development and maintenance. TGF-β, IL-6, and IL-23 promote the differentiation of Th17 cells. TGF-β–induced SMAD and IL-6– and IL-23–mediated STAT3 activation are essential for Th17 cells (4–10). IL-23 is also important for the differentiation of pathogenic T cells producing GM-CSF (11, 12). Signal transducer and activator of transcription 1 (STAT1) is a transcription factor that predominantly mediates signaling downstream of IFN-α, IFN-β, IFN-γ, and IFN-λ. During Th1 differentiation, the transcription factor T-bet, which is weakly induced by TCR stimulation, is further upregulated by IFN-γ/STAT1–mediated signaling (13) and promotes the expression of IL-12Rβ2. Expansion of Th1 cells is then promoted by IL-12–induced STAT4 activation. Consistent with the importance of T cells producing IFN-γ, IL-17, and GM-CSF in the pathogenesis of EAE, mice deficient in the transcription factors T-bet, STAT4, STAT3, RORγt, GM-CSF–promoting factor Bhlhe40, and cytokines GM-CSF, IL-6, IL-23, and IL-23R are protected from EAE development (6, 8, 14–20). In contrast, mice deficient for either IFN-γ, IFN-β, or STAT1 develop exacerbated EAE (18, 21, 22), suggesting a complex role of STAT1 signaling in EAE. Interestingly, type I IFN have been shown to have beneficial effects in Th1 and detrimental ones in Th17-mediated autoimmunity (23, 24). Other studies have postulated that the IFN- and STAT1-mediated protective effects resulted from an inhibition of pathogenic Th17 cells in vitro (7, 25) and enhanced generation of Tregs (26, 27). However, to date, there has been no study testing whether T cell intrinsic STAT1-mediated signaling is necessary in vivo for their pathogenic function in CNS autoimmunity.

Innate lymphoid cells (ILCs) and NK cells are a growing family of immune cells that share phenotypes and functions with Th cells. NK cells evolved to rapidly respond to a variety of insults with cytolytic activity and cytokine secretion (28). These cells are equipped with the lytic machinery to target different cell types. Multiple cytokines have been shown to be crucial for the development, survival, and activation of NK cells, including type I IFN, IFN-γ, IL-2, and IL-15. Notably, IL-2 potently induces perforin and granzyme, which are critical components of NK cell–mediated lysis (29).

NK cells are critical components of the innate immune arm by acting as a main line of defense during infection and tumor growth, recognizing and killing infected and tumor cells, while leaving healthy cells intact (30). Indeed, NK cells have been shown to play a key role in controlling T cell responses, particularly during infection (31–33). However, the impact of NK cells on CNS autoimmunity has been less studied and can change depending on the course of disease progression (34–39). Furthermore, although dysregulation of NK cells has been documented in MS patients (35), limited knowledge exists on the control of autoreactive T cell responses by these cells.

In the present study, we have defined the role of STAT1 in the development of autoreactive Th cell responses and the progression of CNS autoimmunity. Unexpectedly, selective STAT1 deficiency in T cells (STAT1^CD4-Cre^) altered the capacity of CD4^+^ T cells to differentiate into both pathogenic Th1 and Th17 cells and to transfer EAE. While STAT1-deficient T cell proliferation and expansion were normal in vitro, they were profoundly compromised in vivo. We identified NK cells as the active regulatory population limiting autoreactive T cell expansion and EAE development in STAT1^CD4-Cre^ mice. Using adoptive transfers and selective NK cell ablation, we restored the ability of STAT1-deficient CD4^+^ T cells to expand and induce EAE. STAT1 elimination in T cells promoted IL-2 secretion, which directly activated NK cell–mediated cytotoxicity. These results implicate STAT1 as an essential transcription factor for recognition and control of self-reactive CD4^+^ effector T cell responses by NK cells.

## Results

### STAT1 deficiency in CD4^+^ T cells protects mice from EAE development.

STAT1-deficient (STAT1^KO^) mice develop exacerbated EAE compared with control mice (18). To determine if the enhanced disease severity was a result of a skewing of CD4^+^ T cells toward a more pathogenic phenotype, we generated mice with conditional deletion of STAT1 in T cells by crossing STAT1^fl/fl^ mice with CD4-Cre mice (termed STAT1^CD4-Cre^) (Supplemental Figure 1A; supplemental material available online with this article; https://doi.org/10.1172/jci.insight.148222DS1). In these mice, T cells cannot signal through STAT1 in response to IFN-γ or IFN-β (Supplemental Figure 1, B and C). Expectedly, deletion of STAT1 was constrained to T cells, since other cells — such as B cells — were able to respond to IFN-γ and IFN-β in STAT1^CD4-Cre^ mice but not in STAT1^KO^ mice (Supplemental Figure 1, B and C).

**Figure 1:**
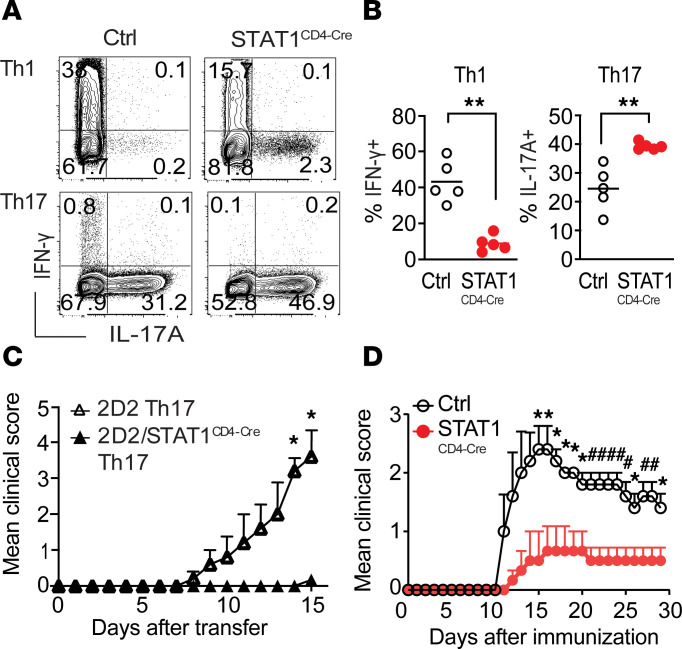
STAT1 deficiency in CD4^+^ T cells protects mice from EAE development. (**A** and **B**) CD4^+^ T cells from WT control and STAT1^CD4-Cre^ mice were differentiated into Th1 or Th17 cells. Representative dot plots show IFN-γ– and IL-17–producing cells among live CD4^+^ T cells (**A**). Plots show the summary of IFN-γ– and IL-17–producing cells among live CD4^+^ T cells (*n* = 5 mice/group) (**B**). Significance calculated with Mann-Whitney *U* test. (**C**) EAE clinical course of recipient mice transferred with MOG-specific 2D2 or 2D2/STAT1^CD4-Cre^ Th17 cells. Results are shown as mean ± SEM over time (*n* = 5–6 mice/group). Significance was calculated with 2-way ANOVA and Fisher’s LSD test. (**D**) EAE clinical course of control STAT1^fl/WT^/CD4-Cre (Ctrl) and STAT1^CD4-Cre^ mice immunized with MOG_35–55_/CFA and pertussis toxin (PT). Results are shown as mean clinical score ± SEM over time (*n* = 5–6 mice per group). Significance was calculated with 2-way ANOVA and Fisher’s LSD test. Data are representative of 2 experiments (*P ≤ 0.05, **P ≤ 0.01, ^#^*P* ≤ 0.01).

Pathogenic CD4^+^ T cells in EAE secrete a combination of IFN-γ, IL-17, and GM-CSF (11, 12). Therefore, we analyzed the capacity of STAT1-deficient T cells to produce these cytokines. After stimulation, similar percentages of CD4^+^ T cells were positive for GM-CSF in WT control mice and STAT1^fl/fl^/CD4-Cre (STAT1^CD4-Cre^) mice (Supplemental Figure 2A). However, we observed differences in cytokine production when T cells from these mice were differentiated into Th1 and Th17 cells to assess IFN-γ and IL-17 production. Although IFN-γ production by Th1 cells was reduced when the cells lacked STAT1, IL-17 expression in both Th1- and Th17-skewed conditions was elevated (Figure 1, A and B). These results are consistent with previous studies illustrating the importance of STAT1 in aiding the differentiation and stability of the Th1 cell phenotype, while repressing the Th17 cell program (7, 13).

The capacity for Th17 cells to be potent inducers of EAE (40, 41) and the enhanced IL-17 production by STAT1-deficient T cells suggested that Th17 cells from STAT1^CD4-Cre^ mice could be more pathogenic than those from control mice. To address this question, we transferred MOG-specific Th17-differentiated 2D2 and 2D2 STAT1^CD4-Cre^ cells into RAG^KO^ recipient mice. Although STAT1-deficient T cells differentiated more efficiently into Th17 cells than WT T cells (Figure 1, A and B), these Th17 cells did not transfer disease as efficiently as WT Th17 cells (Figure 1C), indicating that STAT1 deficiency affected the pathogenic activity of Th17 cells.

To determine whether STAT1 uniquely affected the pathogenicity of Th17 cells, or whether STAT1 deficiency had a broader effect on the development of effector T cell populations, we immunized control and STAT1^CD4-Cre^ mice with MOG_35–55_ emulsified in complete Freund’s adjuvant (CFA), a protocol that generates IFN-γ^+^, GM-CSF^+^, and IL-17^+^ T cells. In stark contrast to STAT1^KO^ mice, STAT1^CD4-Cre^ mice were protected from EAE development (Figure 1D). Contrary to control mice, which had robust and severe EAE, STAT1^CD4-Cre^ mice developed EAE with delayed onset, lower incidence, and less severe clinical signs (Figure 1D). Correlating with disease severity, the numbers of CNS-infiltrating CD4^+^ T cells were significantly reduced in STAT1^CD4-Cre^ mice compared with controls (Supplemental Figure 2B). Therefore, T cells from STAT1^CD4-Cre^ mice, which had diminished IFN-γ, unchanged GM-CSF, and enhanced IL-17 expression, did not induce neuroinflammation.

### Treg depletion and STAT1 deficiency in Foxp3^+^ Tregs does not impact EAE development.

**Figure 2:**
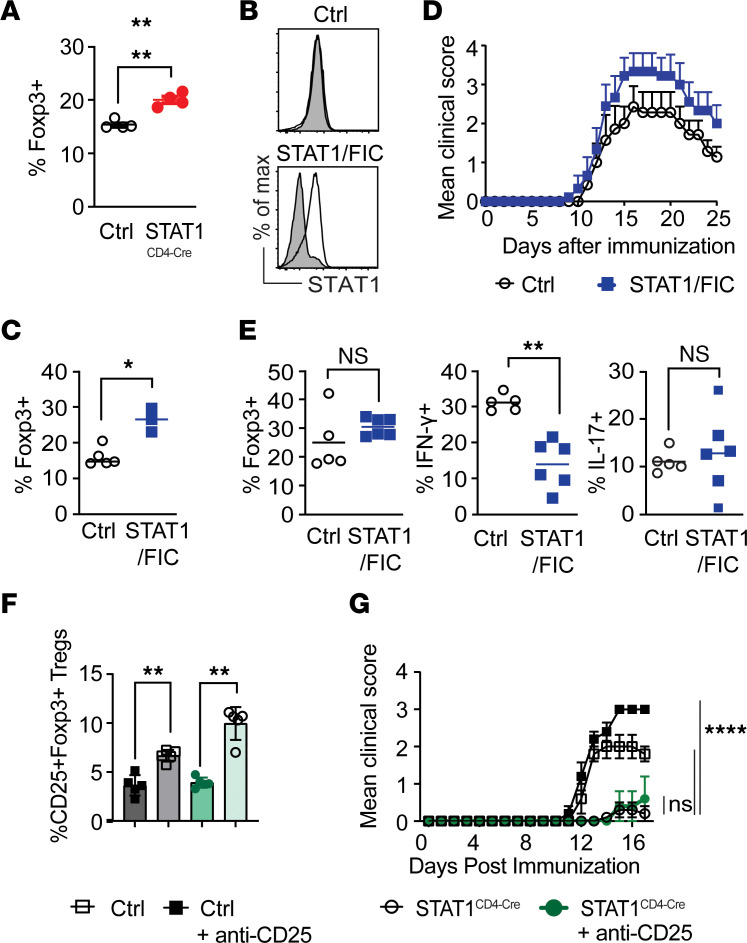
The resistance of STAT1^CD4-Cre^ mice to EAE development is independent of Treg. (**A**) Frequencies of Foxp3^+^ cells in MOG_35–55_–immunized WT control and STAT1^CD4-Cre^ mice (*n* = 4 mice/group). Significance calculated with Mann-Whitney *U* test. (**B**) STAT1 expression in effector CD4^+^Foxp3^–^ (Open histogram) and CD4^+^Foxp3^+^ (Gray-filled histogram) T cells from control (top) or STAT1^fl/fl^/Foxp3-IRES-Cre (STAT1^FIC^) (bottom) mice. (**C**) Frequencies of Foxp3^+^ Tregs in WT and STAT1^FIC^ mice (*n* = 3–5 mice per group). Significance calculated with Mann-Whitney *U* test. (**D**) Clinical scores of WT control and STAT1^FIC^ mice immunized with MOG_35–55_/CFA. Results are shown as mean ± SEM over time (*n* = 7–9 mice per group). Significance was calculated with 2-way ANOVA and Fisher’s LSD test. Data are representative of 2 experiments. (**E**) Summary of Foxp3^+^, IFN-γ^+^ (Th1), and IL-17^+^ (Th17) cells in the CNS of WT and STAT1^FIC^ mice at the end of disease course on day 25 (*n* = 5–6 mice/group). Significance calculated with Mann-Whitney *U* test. (**F**) Percentage of CD4^+^CD25^+^Foxp3^+^ Tregs in the blood of WT and STAT1^CD4-Cre^ mice treated with anti-CD25 or isotype control antibodies, 7 days after immunization with MOG_35–55_/CFA. Data are shown as mean ± SEM (*n* = 5 mice/group). Statistical significance was determined by the Mann-Whitney *U* test. (**G**) Clinical scores of WT control and STAT1^CD4-Cre^ mice, treated with anti-CD25 and immunized with MOG_35–55_/CFA. Data are shown as mean clinical score ± SEM over time (*n* = 5 mice/group). Significance was calculated with 2-way ANOVA with Dunnett’s multiple-comparison test and indicated from day 13 between STAT1^CD4-Cre^ and other groups (*P ≤ 0.05, **P ≤ 0.01, ****P ≤ 0.0001).

Among the different cell types that can limit the pathogenic function of T cells, Foxp3^+^ Tregs play an important role in the control of autoreactive T cells and the impediment of EAE development (42, 43). Furthermore, Treg generation was shown to be altered in STAT1^KO^ mice (26, 27). Therefore, we examined whether STAT1 had a cell-intrinsic effect on Tregs, which could explain EAE resistance in STAT1^CD4-Cre^ mice. After immunization with MOG_35–55_/CFA, we did detect higher frequencies of Foxp3^+^ Tregs in the lymph nodes (LN) of STAT1^CD4-Cre^ mice compared with control mice, suggesting that these cells could be controlling effector T cells (Figure 2A). To determine if STAT1 deletion had a cell-intrinsic effect on Tregs, allowing them to control Foxp3^–^ effector cells more efficiently, we generated mice with selective deletion of STAT1 in Foxp3^+^ T cells (STAT1^fl/fl^/Foxp3-IRES-Cre) termed STAT1^FIC^. The deletion of STAT1 was restricted to Foxp3-expressing cells in the STAT1^FIC^ mice (Figure 2B). Interestingly, similar to STAT1^CD4-Cre^ mice, STAT1^FIC^ mice had higher frequencies of Foxp3-expressing T cells (Figure 2C). The enhanced Treg frequencies in STAT1^CD4-Cre^ and STAT1^FIC^ mice suggested that T cell–intrinsic STAT1 deficiency might promote Treg generation and, therefore, could be responsible for the resistance of STAT1^CD4-Cre^ mice to the development of EAE. To test this hypothesis, we first immunized STAT1^FIC^ for EAE development. However, despite increased frequencies of Tregs in STAT1^FIC^ mice, these mice developed EAE with comparable incidence and severity as control mice (Figure 2D). Although IFN-γ^+^ Th1 cells were reduced in STAT1^FIC^ mice, there was no difference in the frequencies of Th17 and Tregs in the CNS of STAT1^FIC^ mice with EAE (Figure 2E). Therefore, Treg-intrinsic STAT1 deficiency does not significantly modulate EAE course. Next, to address whether Tregs play a role in the resistance of STAT1^CD4-Cre^ mice to the development of EAE, we eliminated Tregs prior to the development of EAE via injection of anti-CD25 antibody. This resulted in a 2-fold reduction in CD25^+^Foxp3^+^ Tregs in controls and STAT1^CD4-Cre^ mice, a week after immunization with MOG_35–55_/CFA/pertussis toxin (PT) (Figure 2F). Consistent with the well-documented ability of Tregs to limit EAE severity in control mice, we observed that control mice treated with anti-CD25 developed exacerbated EAE compared with nontreated mice (Figure 2G). In contrast, elimination of Tregs in STAT1^CD4-Cre^ mice with anti-CD25 antibody treatment did not significantly increase disease severity compared with untreated STAT1^CD4-Cre^ mice (Figure 2G). These data conclusively demonstrate that the resistance of STAT1^CD4-Cre^ mice to the development of EAE is independent of Tregs.

### STAT1 is necessary for CD4^+^ T cell expansion in vivo.

**Figure 3:**
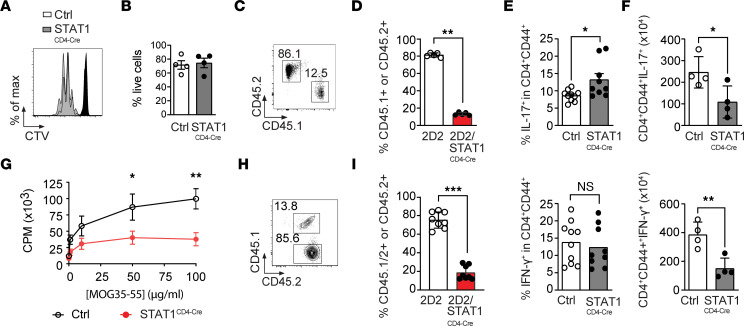
STAT1 is required for T cell proliferation in vivo. (**A**) CTV dilution of CD4^+^ T cells from Ctrl and STAT1C^D4-Cre^ mice stimulated with plate-bound anti-CD3/CD28 for 3 days. Open histogram for WT and gray-shaded histogram for STAT1^CD4-Cre^ mice. (**B**) Percentage of live cells enumerated by flow cytometry. Data are representative of 3 experiments. Significance calculated with 2-tailed Mann-Whitney *U* test. (**C** and **D**) 2D2 (CD45.2^+^) and STAT1^CD4-Cre^/2D2 (CD45.1^+^) CD4^+^ T cells were cotransferred at a 1:1 ratio into RAG^KO^ hosts, which were immunized with MOG_35–55_/CFA and PT. On day 12, CNS were analyzed for the percentage of CD45.2^+^ and CD45.1^+^ cells. Representative (**C**) and quantified (**D**) percentage of CD45.1^+^ or CD45.2^+^ cells among CD4^+^Vβ11^+^ T cells (*n* = 5 mice/group). Significance calculated with 2-tailed Mann-Whitney *U* test. (**E**–**G**) Mice were immunized with MOG35-55/CFA, and 8–10 days later, CD4^+^ T cells from the dLNs were enumerated and analyzed for IFN-γ and IL-17 production. Percentage (**E**) and absolute number (**F**) of cytokine-producing cells among CD4^+^ T cells are presented. Significance was determined by a 2-tailed unpaired *t* test with Welch’s correction (*n* = 4–10 mice/group). (**G**) Proliferative response of dLNs from immunized mice was assessed by [3H] thymidine incorporation in response to MOG_35–55_ peptide. Significance calculated with 2-way ANOVA with Sidak’s multiple comparisons. Data are representative of 2 experiments with *n* ≥ 3 mice in each experiment. (**H**–**I**) Naive CD4^+^ T cells from 2D2 (CD45.2^+^) and STAT1^CD4-Cre^/2D2 (CD45.1^+^CD45.2^+^) mice were cotransferred into RAG^KO^ mice at a 1:1 ratio and immunized with MOG_35–55_/CFA 1 day later. On day 6, frequencies of CD45.2^+^ and CD45.1^+^CD45.2^+^ cells in dLNs were assessed by flow cytometry. Representative (**H**) and quantified (**I**) percentage of CD45^+^ among CD4^+^Vβ11^+^ T cells (*n* = 8 mice/group). Significance calculated with 2-tailed Mann-Whitney *U* test. Data are representative of 2 experiments. (*P < 0.05, **P < 0.01, ***P < 0.001).

STAT1 has been shown to improve the survival and proliferation of lymphocytes (44) but also promote the expression of chemokine receptor and adhesion molecules that could be important for the migration of T cells in the CNS (45–48). Therefore, we next explored if modulation of these pathways could be responsible for the lack of pathogenicity of STAT1-deficient T cells. First, naive CD4^+^ T cells from STAT1^CD4-Cre^ and control mice were sorted by FACS and stimulated with anti-CD3/anti-CD28. There was equivalent proliferation and survival of CD4^+^ T cells from STAT1^CD4-Cre^ and control mice in response to polyclonal stimuli in vitro (Figure 3, A and B).

To address whether STAT1 could be modulating the migration and entry of T cells in the CNS, we cotransferred MOG-specific 2D2 (CD45.2^+^) and 2D2/STAT1^CD4-Cre^ (CD45.1^+^) T cells into RAG^KO^ recipient mice prior to immunization with MOG_35–55_/CFA to induce the development of EAE (Figure 3C). When mice developed EAE (day 12), CNS-infiltrating cells were analyzed to determine the relative frequency of WT 2D2 and STAT1-deficient 2D2 T cells. Significantly fewer STAT1-deficient (CD45.1^+^) CD4^+^ T cells (mean of 13.3% compared with mean of 81.3% for WT) were found in the CNS of mice with EAE (Figure 3, C and D), suggesting that STAT1 expression in T cells is necessary for their presence in the CNS.

Because the analysis of the cotransferred T cells was performed at the peak of the disease, it remained to be determined whether STAT1 selectively affected the migration of T cells in the CNS or had an effect earlier in the expansion of effector T cells prior to their entry in the CNS. To differentiate between these 2 possibilities, STAT1^CD4-Cre^ and control mice were immunized with MOG_35–55_/CFA. Cytokines produced by CD4^+^ T cells in the LN were determined by intracellular cytokine staining. Although the frequencies of Th1 cells were unchanged and the frequencies of Th17 cells were slightly impacted (Figure 3E), we observed an overall decrease in the number of both T cell subsets in STAT1^CD4-Cre^ mice compared with controls (Figure 3F). These results suggest that antigen-specific proliferation and expansion of CD4^+^ T cells might be affected by STAT1 deficiency. Therefore, we next investigated the capacity of STAT1-deficient T cells to proliferate in vivo in response to MOG_35–55_ peptide. STAT1 deficiency in CD4^+^ T cells significantly limited the ability of T cells to respond to the immunizing antigen upon rechallenge, as assessed by thymidine incorporation (Figure 3G), and this was associated with a decrease in the survival of these CD4^+^ T cells (data not shown). Therefore, in the presence of accessory cells, STAT1^CD4-Cre^ T cells do not proliferate efficiently and exhibit decreased survival. This is in sharp contrast with the efficient proliferation observed in response to anti-CD3 and anti-CD28 stimulation (Figure 3A) and the enhanced proliferation that we have previously observed with CD4^+^ T cells from STAT1-deficient mice (18). Therefore, these data suggest that, rather than limiting the trafficking/encephalitogenicity of CD4^+^ T cells, lack of STAT1 expression in T cells might limit their expansion and/or initial priming in the presence of accessory cells.

To test this possibility, we analyzed the distribution of CD4^+^ T cells from 2D2 and 2D2/STAT1^CD4-Cre^ T cells in the draining LN (dLN) of recipient mice 6 days after cotransfer and immunization of RAG^KO^ mice with MOG_35–55_/CFA (Figure 3, H and I). Fewer CD4^+^ T cells from 2D2/STAT1^CD4-Cre^ mice (CD45.1^+^CD45.2^+^) than control 2D2 T cells (CD45.2^+^) (mean of 18.7% versus mean of 75.2%; Figure 3, H, and I) were detected in the LNs of recipient mice. A selective loss of 2D2 STAT1^CD4-Cre^ compared with 2D2 CD4^+^ T cells was also observed when C57BL/6 mice were used as recipient mice instead of RAG^KO^ mice (data not shown), although 2D2 STAT1^CD4-Cre^ T cells survived better in immunocompetent than in immunocompromised mice.

### STAT1 suppresses IL-2 production and protects T cells from NK cell killing.

**Figure 4:**
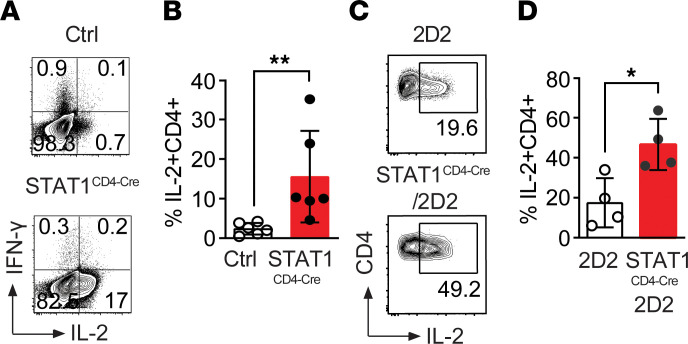
STAT1-deficient CD4^+^ T cells produce more IL-2 than WT CD4^+^ T cells. (**A**) Naive CD4^+^ T cells from Ctrl and STAT1^CD4-Cre^ were stimulated with anti-CD3/CD28 and restimulated with PMA and ionomycin to determine IL-2 production by intracellular cytokine staining on live CD4^+^ T cells. Representative dot plot is shown. (**B**) Summary of IL-2 production by control and STAT1-deficient CD4^+^ T cells stimulated in vitro (*n* = 6 mice/group). Significance was analyzed with Mann-Whitney *U* test. (**C**) IL-2 production from cotransferred 2D2 and STAT1-deficient 2D2 CD4^+^ T cells into RAG1-deficient mice immunized with MOG_35–55_/CFA. (**D**) Quantification of IL-2 production by 2D2 and STAT1-deficient 2D2 cells activated in the same RAG^KO^ host (*n* = 4 mice). Significance was analyzed with Mann-Whitney *U* test (*P ≤ 0.05, **P ≤ 0.01).

The decreased proliferation of STAT1-deficient T cells and lack of expansion in vivo raised the possibility that STAT1-deficient T cells might not respond to IL-2 and/or might produce insufficient amounts of IL-2, which is required for their survival and expansion. Therefore, we tested this possibility. However, against all expectations, upon stimulation, we observed more IL-2^+^ cells in STAT1^CD4-Cre^ than in control CD4^+^ T cells (15.5% versus 2.4 %; Figure 4, A and B). Even more surprising, when control 2D2 and STAT1^CD4-Cre^/2D2 CD4^+^ T cells were cotransferred into RAG^KO^ recipients, STAT1-deficient T cells had more IL-2^+^ cells than control T cells within the same host (46.5% versus 17.5%; Figure 4, C and D). Therefore, STAT1 expression in T cells is important for their expansion and survival after activation in response to MOG_35–55_ peptide and in the presence of innate accessory cells in vivo, and T cell–specific deletion of STAT1 enhances their IL-2 production.

To understand why the in vivo expansion of T cells from STAT1^CD4-Cre^ and STAT1^KO^ mice (18, 44) is so dramatically different, we analyzed the percentage of immune cells present in the lymphoid organs of STAT1^KO^ and STAT1^CD4-Cre^ mice. In addition, we analyzed the expression of different markers on the surface of T cells. While the percentage of TCRβ^+^ T cells was similar between STAT1^KO^ and STAT1^CD4-Cre^ mice, there was a dramatic decrease in the percentage of NK cells in STAT1^KO^ mice compared with STAT1^CD4-Cre^ and control mice (data not shown). This is consistent with the reported dysfunction of NK cells in the STAT1-deficient mice (33, 49, 50), and it suggested to us that NK cells might be involved in the differential phenotype between STAT1^KO^ and STAT1^CD4-Cre^ mice. Since STAT1-deficient T cells produce more IL-2 and common γ chain cytokines, such as IL-2 and IL-15, are strong activators of NK cells (51), we reasoned that STAT1-deficient T cells might affect the phenotype of NK cells via IL-2 and make them more potent at eliminating STAT1-deficient activated T cells.

**Figure 5:**
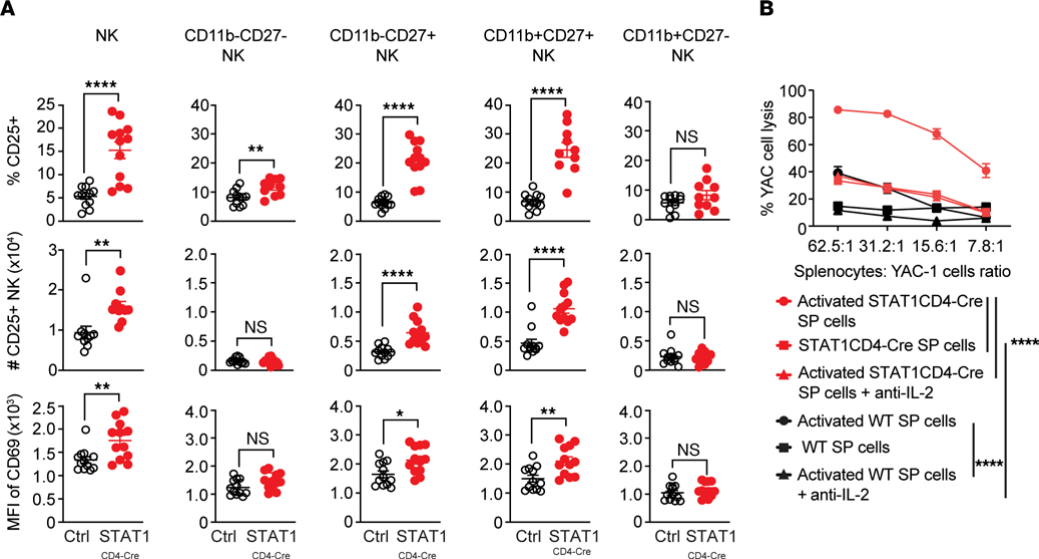
NK cells are activated by enhanced IL-2 expression from STAT1-deficient CD4^+^ T cells and target those cells for cytotoxicity. (**A**) Frequency of lymph node CD25^+^, number of CD25^+^, and MFI of CD69 in (live CD45^+^CD3^–^NK1.1^+^) NK cells, and NK cell subsets (CD11b^–^CD27^–^, CD11b^–^CD27^+^, CD11b^+^CD27^+^, CD11b^+^CD27^–^) from control and STAT1^CD4-Cre^ mice. Data represent individual mouse (*n* = 12/group) and mean group value ± SEM. Significance was analyzed with Mann-Whitney *U* test. (**B**) Killing assay of YAC-1 cells by T cells from WT or STAT1^CD4-Cre^ mice. Spleen cells from WT or STAT1^CD4-Cre^ mice were stimulated or not with anti-CD3/CD28 (in the presence or absence of neutralizing antibodies to IL-2), and 24 hours later, they were added at different ratios to CTV-labeled YAC-1 cells. YAC-1 cell death was quantified at 48 hours by flow cytometry. Data represent the mean percentage of cell death in quadruplicate wells + SD in each condition. Significance was determined by 2-way ANOVA and Tukey multiple-comparison test. Significance is only indicated for selected condition comparisons. Data are representative of 3 independent experiments (*P ≤ 0.05, **P ≤ 0.01, ****P < 0.0001).

First, we assessed whether NK cell phenotype was indeed altered in STAT1^CD4-Cre^ mice. We analyzed the expression of CD25 (IL-2Ra) and CD69 on LN NK cells from control and STAT1^CD4-Cre^ mice. Interestingly, we observed more CD25^+^ NK cells in STAT1^CD4-Cre^ mice compared with control mice (Figure 5A). Furthermore, NK cells from STAT1^CD4-Cre^ mice had higher expression of the activation marker CD69 than control mice (Figure 5A). Surface density of CD27 and CD11b subdivides mouse NK cells into 4 subsets: CD11b^–^CD27^–^, CD11b^–^CD27^+^, CD11b^+^CD27^+^, and CD11b^+^CD27^–^. Therefore, we looked at whether STAT1^CD4-Cre^ NK cell differential phenotype occurred in all subsets or was more selective. Consistently, CD25^+^CD11b^–^CD27^+^ and CD25^+^CD11b^+^CD27^+^ NK cells subsets were selectively increased, and these subsets also expressed more CD69 in STAT1^CD4-Cre^ mice (Figure 5A). Therefore, NK cells from STAT1^CD4-Cre^ mice are more activated and poised to respond to IL-2.

Next, we determined whether IL-2 production by STAT1-deficient CD4^+^ T cells activated NK cells and led to enhanced NK cell lytic activity. We activated T cells in NK cell–containing spleen cell cultures from control and STAT1^CD4-Cre^ mice and tested the capacity of NK cells to lyse YAC-1 target cells in the presence or absence of an anti–IL-2 neutralizing antibody (Figure 5B). We observed that activated STAT1-deficient CD4^+^ T cells enhanced the activation of WT NK cells in these cultures and their capacity to lyse YAC-1 target cells (85.6% of target cells lysis versus 39% when NK cells were activated with WT T cells). When IL-2 was blocked, the killing of YAC cells in STAT1^CD4-Cre^ cell cultures was diminished to levels observed in WT cell cultures, supporting the fact that the enhanced IL-2 secretion by STAT1^CD4-Cre^ T cells promotes the killing activity of NK cells toward STAT1^CD4-Cre^CD4^+^ T cells (Figure 5B). Therefore, we have established that STAT1-deficient T cells, through their increased production of IL-2, enhanced the activity of NK cells to lyse them in vitro. Next, we wanted to determine whether this phenomenon could also act in vivo and limit EAE development in STAT1^CD4-Cre^ mice.

### NK cells selectively target STAT1-deficient CD4^+^ T cells, limiting EAE development.

**Figure 6:**
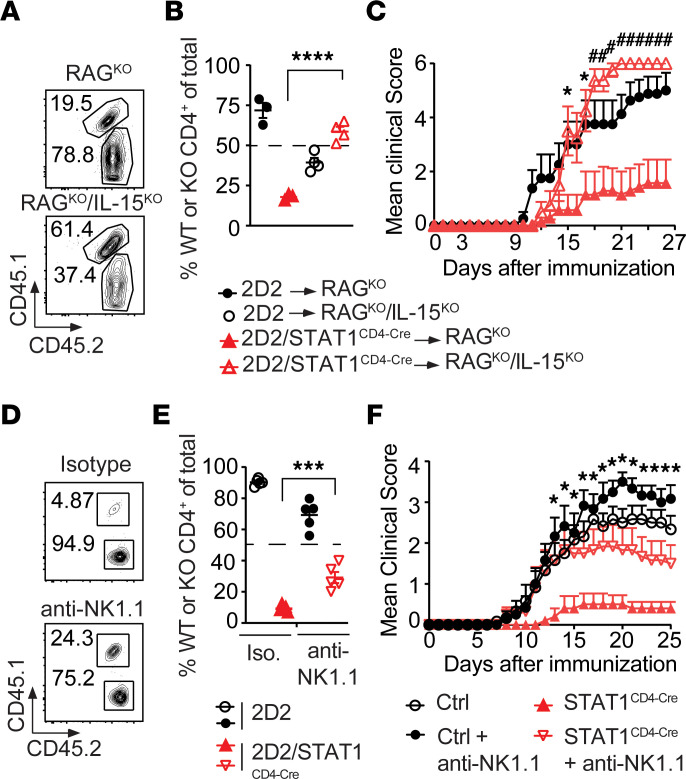
NK cells selectively target STAT1-deficient CD4^+^ T cells for elimination and suppress EAE. (**A** and **B**) Congenically marked CD4^+^ T cells from 2D2 (CD45.2^+^) and STAT1^CD4-Cre^/2D2 (CD45.1^+^CD45.2^+^) were cotransferred into RAG^KO^ and RAG^KO^IL-15^KO^ mice at a 1:1 ratio, followed by immunization with MOG_35–55_/CFA. Six days after transfer, the draining lymph nodes were harvested. Frequencies (**A**) and quantification expressed as the percentage of total CD4^+^ T cells (**B**) of WT 2D2 (CD45.2^+^) and STAT1-deficient 2D2 (CD45.1^+^CD45.2^+^) donor CD4^+^ T cells, assessed by flow cytometry. Significance was calculated with 2-way ANOVA with Sidak’s multiple-comparison test (*n* = 3–4 mice per group). (**C**) CD4^+^ T cells from 2D2 and STAT1^CD4-Cre^/2D2 were transferred into RAG^KO^ and RAG^KO^IL-15^KO^ mice 1 day before immunization with MOG_35-55_/CFA — and treatment with pertussis toxin at the time of immunization — and 2 days later. Clinical scores of mice are shown. Results are shown as mean ± SEM over time. Significance was calculated with 2-way ANOVA with Dunnett’s test (*n* = 7–8 mice/group). (**D** and **E**) Congenically marked naive CD4^+^ T cells from 2D2 (CD45.2^+^) and STAT1^CD4-Cre^/2D2 (CD45.1^+^CD45.2^+^) were cotransferred into anti-NK1.1 mAb– or isotype control–treated RAG^KO^ mice at a 1:1 ratio, followed by immunization with MOG_35–55_/CFA. Six days after transfer, the draining lymph nodes were harvested. Frequencies (**D**) and quantification (**E**) of WT (CD45.2^+^) and STAT1-deficient (CD45.1^+^CD45.2^+^) donor CD4^+^ T cells, assessed by flow cytometry. Significance was calculated with 2-way ANOVA with Sidak’s multiple-comparison test. Data are representative of 2 experiments with *n* = 5 mice/group. (**F**) Effects of anti-NK1.1 mAb treatment on the development and progression of EAE in control and STAT1^CD4-Cre^ mice. Results are shown as mean ± SEM over time. Significance was calculated with 2-way ANOVA with Fisher’s LSD test (*n* = 10–12 mice per group). Data are representative of 2 experiments (*P ≤ 0.05, **P ≤ 0.01, ^#^*P* ≤ 0.01, ***P ≤ 0.001, ****P ≤ 0.0001).

To test this hypothesis, we took advantage of the fact that IL-15 is an important survival factor for NK cells and that NK cell activity is defective in IL-15–deficient RAG^KO^ (RAG^KO^IL-15^KO^) mice (52). Therefore, we cotransferred naive cells from 2D2 (CD45.2^+^) and STAT1^CD4-Cre^/2D2 (CD45.1^+^CD45.2^+^) mice into RAG^KO^IL-15^KO^ recipient mice to address the role of NK cells in the lack of expansion of STAT1-deficient T cells. As control, 2D2 and STAT1^CD4-Cre^/2D2 naive T cells were cotransferred into RAG^KO^ mice. Consistent with our previous results (Figure 3, H and I), 2D2 T cells transferred into RAG^KO^ mice expanded, while STAT1^CD4-Cre^/2D2 expanded 4-fold less (Figure 6, A and B). However, similar percentages of 2D2 and STAT1^CD4-Cre^/2D2 T cells were recovered if these cells were cotransferred into RAG^KO^IL-15^KO^ mice in which NK cell activity was compromised (Figure 6, A and B). To test if the control of T cell expansion was a limiting factor in their capacity to induce EAE, STAT1-deficient 2D2 as well as WT 2D2 CD4^+^ T cells, were transferred into RAG^KO^ and RAG^KO^IL-15^KO^ mice and EAE was induced with MOG_35–55_/CFA and PT. When transferred into RAG1-deficient mice, 2D2 CD4^+^ T cells induced severe EAE. Consistent with limited expansion of STAT1^CD4-Cre^/2D2 T cells in RAG^KO^ without PT injection (Figure 6, A and B), the transfer of STAT1^CD4-Cre^/2D2 cells in RAG^KO^ with PT induced limited EAE development (Figure 6C). However, when CD4^+^ T cells from STAT1^CD4-Cre^/2D2 mice were transferred into RAG^KO^IL-15^KO^ mice, which have compromised NK cell activity, EAE severity was restored and was equivalent to the disease observed in RAG^KO^ mice transferred with 2D2 T cells (Figure 6C), suggesting that NK cells suppress STAT1-deficient T cells and prevent them from inducing EAE.

Because IL-15 deficiency might interfere with the antigen presenting capacity of DCs in addition to NK cells, we used a complementary approach to establish the involvement of NK cells in the elimination of activated STAT1-deficient CD4^+^ T cells. Congenically marked 2D2 (CD45.2^+^) and STAT1^CD4-Cre^/2D2 (CD45.1^+^CD45.2^+^) T cells were cotransferred into RAG^KO^ hosts, which were subsequently immunized with MOG_35–55_. NK cells were depleted by injection of an anti-NK1.1 antibody or left untouched in a group of mice treated with isotype control. In isotype-treated mice, while WT 2D2 cells represented close to 95% of the total CD4^+^ T cells, T cells from STAT1^CD4-Cre^/2D2 mice accounted for only 5% of all CD4^+^ T cells (Figure 6, D and E). The frequency of STAT1^CD4-Cre^/2D2 T cells was significantly increased (from 9.1% to 29.2%) when recipient mice were treated with an anti-NK1.1 antibody but remained lower than the frequency of 2D2 T cells. To determine if the elimination of NK cells resulted in the consequential increase in STAT1-deficient CD4^+^ T cells, we immunized controls and STAT1^CD4-Cre^ mice with MOG_35–55_/CFA and PT to induce EAE, and we treated half of them with an anti-NK1.1 antibody to deplete NK cells and half of them with an isotype control. Consistent with previous literature (39), depletion of NK cells in WT mice induced more severe EAE than in isotype-treated mice. Importantly, while STAT1^CD4-Cre^ mice were resistant to EAE development, as described in Figure 1, STAT1^CD4-Cre^ mice treated with anti-NK1.1 developed EAE, with severity equivalent to that observed in control mice (Figure 6F). Therefore, NK cells limit the expansion and number of MOG-reactive STAT1-deficient CD4^+^ T cells and, thus, prevent the development of EAE.

## Discussion

In summary, we have established here that STAT1^CD4-Cre^ mice are resistant to the development of EAE, a phenotype that contrasts with STAT1^KO^ mice, which are highly sensitive to the development of EAE (18). In addition, while CD4^+^ T cells from STAT1^CD4-Cre^ mice produced more IL-17 and had more Foxp3^+^ Tregs, Th17 cells from these mice failed to induce robust EAE, and STAT1 deletion in Foxp3^+^ T cells did not limit EAE development. Instead, we have established that NK cells represent another regulatory mechanism controlling the expansion of a myelin-autoreactive response, which may not be fully effective or dominant in WT mice but is selectively and strongly induced in STAT1^CD4-Cre^ mice. Indeed, we showed that STAT1-deficient T cells produce more IL-2 than WT T cells, thus promoting NK cell lytic activity against them.

Tregs have predominantly been implicated in the regulation of CD4^+^ T cell responses. The contribution of IFN- and STAT1-mediated signaling for Treg homeostasis and function has been contradictory and may be context dependent. On the one hand, type I IFN maintain Foxp3 expression and Treg functions (53, 54). On the other hand, a suppressive effect of IFN-γ–, IFN-α–, and STAT1-mediated signaling on Tregs has also previously been reported (26, 27, 55, 56). In our study, ablation of STAT1 in T cells or in Foxp3^+^ Tregs led to an increase in the frequency of Foxp3^+^ T cells in mice. However, against all expectations, the increased frequency of Tregs in STAT1^CD4-Cre^ and STAT1^FIC^ mice did not protect mice actively immunized for the development of EAE (Figure 2). Instead, while T cell activation, proliferation, and survival were intact in STAT1-sufficient and -deficient T cells primed in vitro, we established that these cells failed to expand when cotransferred into RAG1-deficient recipients and after activation with their cognate antigen in vivo, revealing an in vivo–specific and accessory cell–dependent effect of STAT1 abrogation (Figures 3 and 4).

Here, we have identified a regulatory mechanism independent of Tregs but dependent on NK cells by which CD4^+^ T cell responses can be controlled during CNS autoimmunity. Control of effector T cell responses by NK cells has been observed in the context of infection (31, 33, 57) and has been suggested in autoimmunity (58, 59), but direct evidence for NK cells affecting autoreactive myelin-specific CD4^+^ cells has not been described. We have demonstrated that selective deletion of STAT1 in T cells promotes an essential regulatory mechanism mediated by NK cells, resulting in the elimination of effector T cells. The lack of EAE development in STAT1^CD4-Cre^ mice contrasts with the enhanced EAE susceptibility of STAT1^KO^ mice (18). One reason for these differences is likely explained by the profound effect of STAT1 on NK cell biology. Indeed, consistent with previous reports (49, 50), we have observed that STAT1-deficient mice have reduced NK cell numbers. Furthermore, STAT1 promotes NK cell survival and cytotoxic activity (49, 50), since STAT1-deficient mice have impaired NK cell responses to vaccinia virus (50) and since STAT1-mediated type I IFN signaling is required for an optimal antiviral NK cell response (33). Therefore, unlike STAT1^CD4-Cre^ mice, which harbor functional WT NK cells, NK cells from STAT1^KO^ mice are sparse and defective, and they fail to control effector T cells.

A role for NK cells in regulating autoimmunity, particularly MS and its animal model EAE, has also emerged (35). While some reports propose a pathogenic function (60, 61), most studies have shown that NK cells have a protective role in EAE (39, 62–64). Our data support their protective role in EAE since depletion of NK cells with an anti-NK1.1 antibody exacerbated EAE development in both WT and STAT1^CD4-Cre^ mice (Figure 6F). Although NK1.1 is also expressed by ILC1, our experiments showing strong EAE development in RAG^KO^IL-15^KO^ mice, which have ILC1 but lack NK cells compared with RAG^KO^ mice, transferred with STAT1^CD4-Cre^/2D2 CD4^+^ T cells (Figure 6C) further support the involvement of NK cells, and not ILC1, in controlling MOG specific CD4^+^ T cell responses. Several studies have shown impaired NK cell function and numbers in MS patients with active lesions (59, 65–68), as well as the existence of transient valleys of NK cell function that manifested during relapses (65). Interestingly, both Treg- and NK cell–mediated mechanisms of regulation are compromised in MS patients. Currently, the details of their suppressive activity are unclear, and it would be worth investigating whether these 2 regulatory mechanisms act in concert or at different time points during EAE and MS development and progression. Alternatively, these 2 mechanisms of regulation might be competing with one another. In support of this hypothesis, Rudensky and colleagues demonstrated that Tregs restrain the IL-2–dependent CD4^+^ T cell help for CD127^+^ immature NK cells and NK cell cytotoxicity by limiting the availability of IL-2 (69, 70). Our data support the hypothesis that T cell–derived IL-2 expands immature CD25^+^CD27^+^CD11b^–^ and CD25^+^CD27^+^CD11b^+^ NK cells subsets and that these subsets are more poised to respond to IL-2 via their expression of CD25 in STAT1^CD4-Cre^ mice. In STAT1^CD4-Cre^ mice whose T cell produce more IL-2, our data further demonstrate that NK cell–mediated killing of autologous T cells occurred independently of regulation by Treg (Figure 2G).

A balance between activating and inhibitory signals orchestrates and dictates the outcome of NK cell function. Recognition of MHC class I molecules on the surface of healthy cells from self-tissues help prevent against targeting by NK cells and other cells which lack MHC-I, such as tumors, can be targeted by NK cells (71). However, the lack of MHC class I does not appear to be sufficient to trigger cell lysis by NK cells (72). The upregulation of activating receptors and the activation of NK cells by cytokines, such as type I IFN, IFN-γ, IL-2, IL-12, IL-15, and IL-18, are necessary for NK cells to lyse their targets (73–75). Here, we have established that STAT1-deficient T cell enhanced IL-2 production promoted NK cell activation and lysis of CD4^+^ T cells (Figure 5, A and B). The importance of IL-2 for the activation NK cells is supported by reports showing that activated T cell–derived IL-2 can activate NK cells in vitro (76). Interestingly, IL-2 was shown to activate ERK, but not p38 MAPK, in NK lymphocytes without prior stimulation (77). IL-2 acts in a paracrine manner, and its half-life is extremely short (78). Therefore, cells that produce the highest amount of IL-2 (i.e., from 2D2/STAT1^CD4-Cre^ mice in our study) are likely to be preferentially targeted by NK cells in their vicinity, a phenomenon that we have observed in our cotransfer experiments (Figure 3, D and I, and Figure 6, B and E). However, additional signals may also enhance T cell killing by NK cells. Modulation of the IL-2 receptor with daclizumab has been shown to enhance the cytolytic activity of NK cells and to increase the expression of CD155-expressing CD4^+^ T cells, thus rendering CD4^+^ T cells likely more sensitive to NK-mediated lysis through DNAM-1 (59). Furthermore, previous reports determined that type I IFN signaling protected CD8^+^ T cells killing by NK cells during the course of LCMV infection (79, 80). Indeed, IFNAR-deficient CD8^+^ T cells were lysed by activated NK cells, and this process was shown to be dependent on NCR1 (80). Therefore, whether NCR1 is also involved in the killing of STAT1-deficient CD4^+^ T cells and whether IL-2 could upregulate NCR1 ligands would be of interest to determine in future studies.

While NK-mediated cytotoxicity toward T cells is enhanced in STAT1^CD4-Cre^ mice compared with control mice due to enhanced IL-2 production by T cells, we propose that there is a dynamic baseline level of NK cell–mediated cytotoxicity toward T cells in WT animals, as well. For the most part, NK cells are “educated” not to kill autologous T cells, but this is not an absolute phenomenon. Indeed, published data show that NK cells are capable of killing activated MHC-I^+^ T cells (32, 81, 82). We have made similar observations in vivo (Supplemental Figure 3 and data not shown). Therefore, NK cells have the capacity to and do eliminate autologous T cells with MHC-I expression, but this phenomenon is likely to be relatively rare in immunocompetent WT mice because IL-2 production is limited and can be further used by CD25 expressing Treg. In contrast, we have established that NK cells from STAT1^CD4-Cre^ mice have more CD25^+^ NK cells and are, therefore, poised to respond to limited amount of IL-2. Considering that STAT1^CD4-Cre^ T cells produce more IL-2 and that IL-2 acts in a very paracrine manner (78), this enhances the capacity of NK cells to kill autologous T cells in STAT1^CD4-Cre^ mice. We propose that the ability of certain IL-2 activated NK cells to kill autologous T cells could be a regulatory mechanism in place to limit the expansion of overly activated T cells. This mechanism would be effective in circumstances when IL-2 is more readily available and Tregs are less effective or outnumbered by effector T cells (i.e., inflammation).

Therefore, while it is well established that NK cells play a critical role in cancer immunity and tumor surveillance, our studies have uncovered an unexpected immunoregulatory role for NK cells, which is to prevent autoimmunity by limiting the expansion of autoreactive T cells.

## Methods

### Mice.

C57BL/6 (CD45.2), B6/SJL (CD45.1), CD4-Cre, and RAG1^KO^ mice were purchased from the Jackson Laboratory and bred in the Benaroya Research Institute (BRI) animal facility. STAT1^fl/fl^ mice have been described previously (83) and were provided by Daniel J. Campbell at the Benaroya Research Institute at Virginia Mason, Seattle, Washington, USA. They were crossed with either FIC mice (84) or CD4-Cre mice (The Jackson Laboratory) to generate STAT1^FIC^ or STAT1^CD4-Cre^ mice. Control mice used for STAT1^CD4-Cre^ mice in the present study were either STAT1^fl/WT^/CD4-Cre or C57BL/6 mice. 2D2 TCR transgenic mice have been previously described (85). RAG^KO^IL-15^KO^ mice were provided by Mohamed Oukka (University of Washington, Seattle, Washington, USA). All strains were on the C57BL/6 background, and male and female mice were used. All animals were bred and maintained under specific pathogen-free conditions at the BRI.

### Depletion of Tregs.

For in vivo depletion of Tregs, mice were injected i.p. with either 500 μg/mouse of anti-CD25 (PC61, Bio X Cell) or isotype control rat IgG1 antibodies, 3 days and 1 day prior to immunization with MOG35-55/CFA emulsion and PT. Mice were bled on day 7 after immunization to check Treg depletion efficacy.

### Depletion of NK cells.

For depletion of NK cells, mAb to NK1.1 (PK136, Bio X Cell) was injected at 200 μg/mouse 1 day prior to the transfer of CD4^+^ T cells and every 3–4 days subsequently. In active EAE, control and STAT1^CD4-Cre^ mice received 200 μg/mouse of NK1.1 mAb 1 day before immunization with MOG_35–55_ and every 3–4 days subsequently.

### CD4^+^ T cell preparation and T cell differentiation.

For T cell differentiation, naive CD4^+^CD62L^hi^CD44^lo^CD25^−^ T cells were isolated by FACS (FACSAria, BD Biosciences) and cultured with irradiated (4000 rads) CD4-depleted spleen cells from WT mice and anti-CD3 (2.5 μg/mL, clone 145-2C11) for 5 days in complete RPMI. For Th1 differentiation, 10 ng/mL rmIL-12 was added.

For Th17 differentiation, 2.5 ng/mL rhTGF-β (R&D Systems, catalog 240-B-010), 30 ng/mL rmIL-6 (Peprotech, catalog 216-16), 10 µg/mL anti–IFN-γ (Bio X cell, catalog BE0055, clone XMG1.2), and 10 µg/mL anti–IL-4 (NIH/NCI BRB Preclinical Repository, catalog 50184, clone 11B11) were used. For transfer experiments, IL-23 (R&D Systems, catalog 1887-ML) was also added, and anti–IFN-γ was excluded from the Th17 cultures

### Antibodies, flow cytometry, and cell sorting.

For surface staining, cells were incubated at 4°C for 30 minutes in staining buffer (PBS, 2% FCS) with the following antibodies directly conjugated to fluorochromes from BioLegend: CD4 (clone GK1.5), CD44 (clone 1M7), CD25 (clone PC61), CD62L (clone MEL-14), CD45.2 (clone 104), CD45.1 (clone A20), CD45 (clone 30-F11), CD3 (clone 17A2), NK1.1 (clone PK136), CD69 (clone H1.2F3), CD11b (clone M1/70), and CD27 (clone LG 3A10); from eBioscience, the following antibodies were used: CD4 (clone GK1.5), CD62L (clone MEL-14), CD69 (clone H1.2F3), and CD11b (clone M1/70). For intracellular cytokine staining analysis, cells were incubated 5 hours in complete RPMI containing 50 ng/mL phorbol myristate acetate (PMA), 1 μg/mL ionomycin (Sigma-Aldrich), and Golgi Stop (BD Biosciences). Cells were then washed with cold PBS and blocked for 10 minutes with anti-CD16/32 purified antibody (2.4G2, Bio X Cell). Viability of the cells was assessed by staining with fixable dye eFluor780 (eBioscience). Cells were then stained with surface antibodies and washed with PBS. Cells were then fixed for 20 minutes with fixation buffer (BD Biosciences), permeabilized with BD permeabilization/wash buffer (BD Biosciences), and stained with anti–IFN-γ (clone XMG1.2, BioLegend), and anti–IL-17 (clone TC11-18H10.1, BioLegend) specific antibodies or anti–IL-2 specific antibodies (JES6-5H4, BioLegend) in permeabilization buffer. Cells were acquired on LSRII (BD Biosciences), and data were analyzed with FlowJo software.

### Proliferation assays.

For in vitro proliferation assays, naive CD4^+^ T cells were labeled with either 5 μM CFSE (eBioscience) or 5 μM Cell Trace Violet (CTV; Molecular Probes) for 10 minutes at 37°C in PBS, washed with complete RPMI, and stimulated with 2.5 µg plate-bound anti-CD3 (Bio X Cell, catalog BE0001-1, clone 145-2C11) and anti-CD28 (Bio X Cell, catalog BE0015-5, clone PV-1) for 3 days. For in vitro recall responses of MOG-specific cells, dLN of immunized mice were collected 8–10 days after immunization with MOG_35–55_/CFA. Cells were cultured at 5 × 10^6^ cells/mL in the presence of different concentrations of MOG_35–55_ for 72 hours. During the last 16 hours, cells were pulsed with 1 μCi [^3^H]thymidine. [^3^H]thymidine incorporation was measured using a β-counter.

### NK cytotoxicity assays.

Splenocytes were stimulated with Dynabeads Mouse T-activator CD3/CD28 (Thermo Fisher Scientific) to activate T cells for 24 hours in the presence or absence of anti–mouse IL-2 antibodies at 20 μg/mL (clones JES6-1A12 and S4B6-1, Bio X Cell) at 37°C in cell culture medium (RPMI 1640; Thermo Fisher Scientific, catalog 11875093) supplemented with 5% FCS (VWR, catalog 97068-085), penicillin-streptomycin (Thermo Fisher Scientific, catalog 15140122), and β-mercaptoethanol (Thermo Fisher Scientific, catalog 21985023). After 24 hours, target YAC-1 cells were labeled with 5 μM CTV (Thermo Fisher Scientific). In total, 2 × 10^4^ CTV labeled YAC-1 cells were added at different ratios to stimulated splenocytes. At 48 hours (24 hours of coculture of target YAC-1 cells with stimulated splenocytes), cells were harvested and incubated with Viability Dye eFluor (eBioscience, Invitrogen) for 10 minutes at room temperature in the dark. Cells were washed once with PBS and then analyzed on BD LSR II. The percentage of dead cells within CTV^+^ YAC-1 cells was quantified. The percentage of lysis represents the percentage of dead YAC-1 cells in coculture of YAC-1 cells and spleen cells minus the percentage of dead YAC-1 cells in culture of YAC-1 cells alone.

### Adoptive transfer of CD4^+^ T cells.

For cotransfer experiments 0.5 × 10^6^ sorted naive CD4^+^ T cells from congenically marked control 2D2 and STAT1^CD4-Cre^/2D2 mice were transferred into RAG^KO^ mice 1 day prior to immunization with 50 μg/mouse of MOG_35–55_ emulsified in CFA.

### EAE induction.

EAE was induced by s.c. immunization of mice into the flanks with an emulsion of MOG_35–55_ peptide (150 μg) emulsified in complete Freund adjuvant supplemented with 4 mg/mL of *M*. *tuberculosis* extract H37Ra (Difco). In addition, the animals received 200 ng of PT (List Biological Laboratories) i.p. on days 0 and 2. Clinical signs of EAE were assessed according to the following score: 0, no signs of disease; 1, loss of tail tonicity; 2, righting reflex; 3, partial hind limb paralysis; 4, complete hind paralysis; 5, complete hind and forelimb paralysis; and 6, moribund.

### Passive EAE induction.

In total, 50,000 naive CD4^+^ T cells from 2D2 and STAT1^CD4-Cre^/2D2 mice were transferred into RAG1^–/–^ mice and immunized s.c. with 25 μg MOG_35–55_ in CFA, and 200 ng PT was injected i.p. the same day and 2 days after. For transfer of Th17 cells, sorted naive CD4^+^ T cells from 2D2 and STAT1^CD4-Cre^/2D2 mice were differentiated in vitro for 4 days in the presence of anti-CD3, irradiated APCs, and IL-23, as described above, and 5 × 10^6^ cells from each mouse genotype (2D2 and STAT1^CD4-Cre^/2D2) were transferred into RAG^KO^ mice with PT (200 ng) injected the same day and 2 days later. Clinical signs of EAE were assessed according to the following score: 0, no signs of disease; 1, loss of tail tonicity; 2, righting reflex; 3, partial hind limb paralysis; 4, complete hind paralysis; 5, complete hind and forelimb paralysis; and 6, moribund.

### Isolation of CNS mononuclear cells.

Mice were sacrificed at the peak of disease and perfused with cold PBS. Brain and spinal cords were isolated and digested for 30 minutes at 37°C with Collagenase D at a concentration of 2.5 mg/mL (Roche). Mononuclear cells were isolated over a 37%/70% Percoll gradient (VWR), washed twice with complete medium, and collected in medium for further analysis.

### Statistics.

Statistical analyses were conducted with GraphPad Prism software. Clinical scores were compared using 2-way ANOVA with Dunnett’s and Fisher’s LSD post hoc tests. The percentages of differentiated Th cells, cytokine producing Th cells, and Foxp3^+^ Tregs were compared using the 2-tailed Mann-Whitney *U* test. Absolute numbers of cells were compared with 2-tailed unpaired *t* test with Welch’s correction. Proliferation was compared with 2-way ANOVA with Sidak’s multiple comparisons. Percentage of lysis was compared with 2-way ANOVA and Tukey multiple-comparison test. *P* values of less than 0.05 were considered significant.

### Study approval.

All experiments were performed by the guidelines of the BRI Animal Care and Use Committee.

## Author contributions

CAA performed most of the initial experiments and wrote the manuscript with EB. PP performed additional experiments, including but not limited to in vitro killing assays, Treg depletion, and NK phenotyping, that were necessary for the revisions. She also contributed to the revised final figures. JC performed supplemental experiments (histology, transfer) to address the reviewers’ comments. EB designed and supervised the study, wrote the manuscript, and made the figures.

## Supplementary Material

Supplemental data

## Version 1 05/19/2022

In-Press Preview

## Version 2 06/22/2022

Electronic publication
