# Supplementary material for: STAT1 signaling protects self-reactive T cells from control by innate cells during neuroinflammation
Source: JCI Insight. 2022 Jun 22;7(12):e148222. doi: 10.1172/jci.insight.148222 (PMC9309063; doi:10.1172/jci.insight.148222)
Supplement: Supplemental data [file jciinsight-7-148222-s162.pdf]

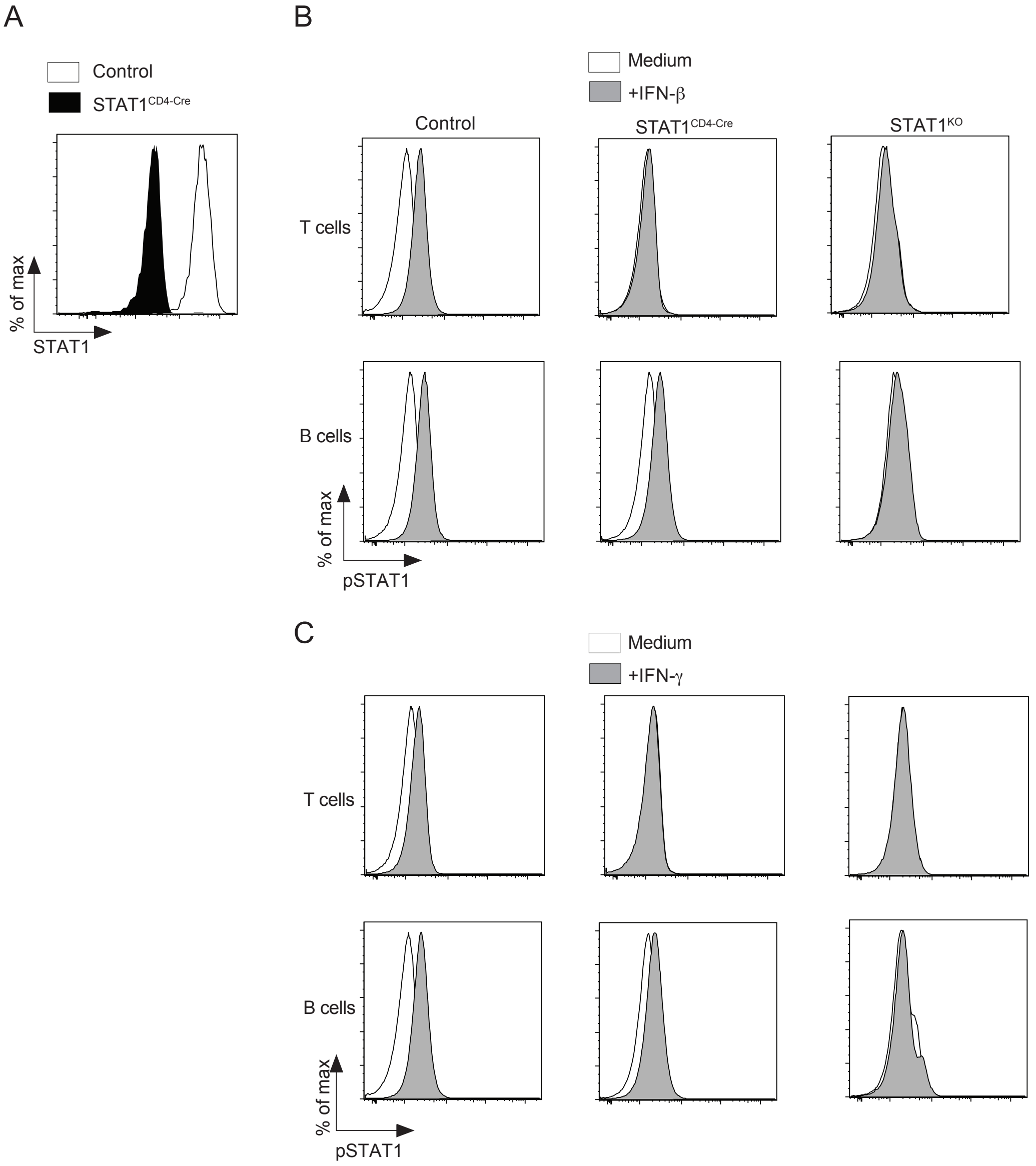

Supplementary figure 1. STAT1 expression. (A) STAT1 expression in splenic T cells from Controls (Open histogram) and STAT1$^{CD4\text{-}Cre}$ mice (Black filled histogram). **(B-C)** Phospho-STAT1 expression in T cells and B cells from control, STAT1$^{CD4\text{-}Cre}$, and STAT1^{KO} mice stimulated (Gray filled histogram) or not (Open histogram) with IFN-β (B) or IFN-g (C) for 30 minutes.

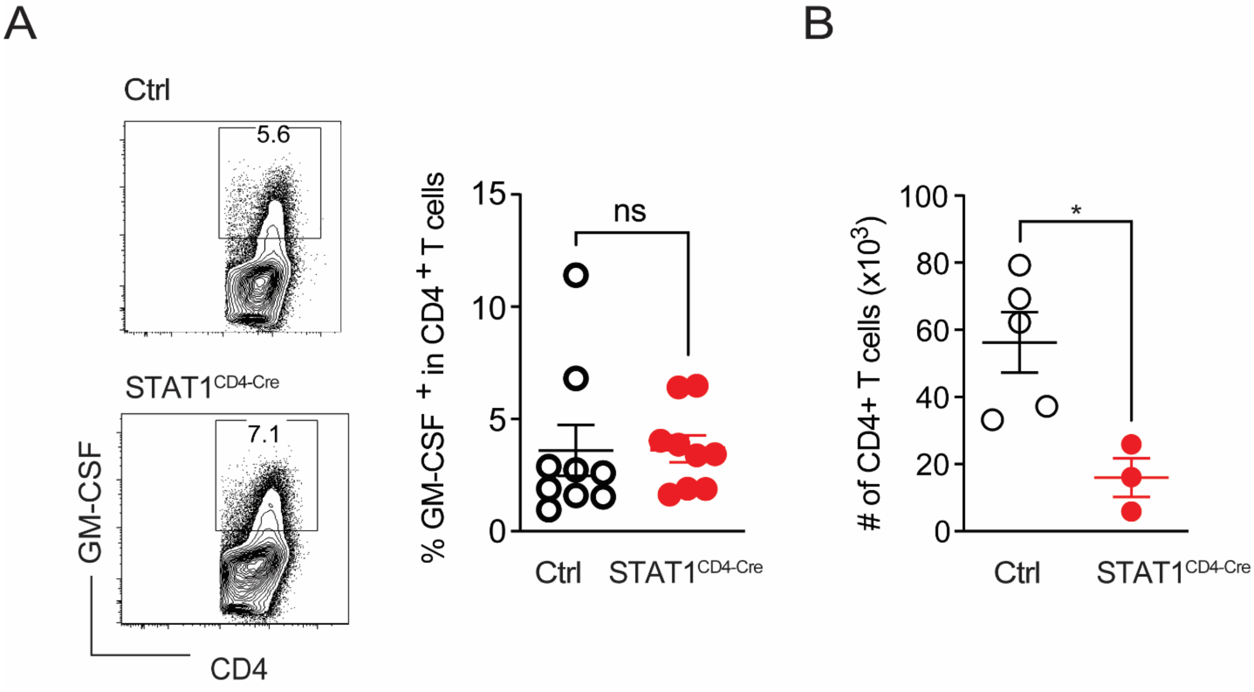

Supplementary figure 2. CD4+ T cells in STAT1$^{CD4\text{-}Cre}$ and control mice. (**A**) Representative dot plots show GM-CSF producing cells among live CD4+ T cells of CD4+ T cells from WT control and STAT1$^{CD4\text{-}Cre}$ mice stimulated with anti-CD3 and antigen-presenting cells (APCs) in non-polarizing conditions (Left). Box graph shows the summary of GM-CSF producing cells among live CD4+ T cells (n=9 mice/group) (Right). Significance calculated with two-tailed unpaired t test with Welch's correction. **(B)** Numbers of CD4+ T cells isolated from the brain and spinal cord (CNS) of control and STAT1$^{CD4\text{-}Cre}$ mice which developed EAE, were quantified at the end of the disease (n=3-5 mice/group). Significance calculated with two-tailed Mann-Whitney test. Data are representative of 2 experiments ($^{*}p < 0.05$).

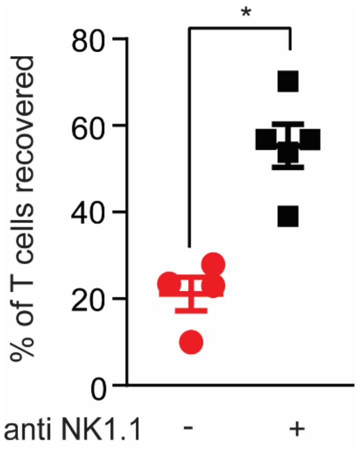

Supplementary figure 3. NK-mediated deletion of activated T cells in vivo. Negatively selected splenic C57BL/6 CD44- naïve T cells were *in vitro* activated with anti-CD3/CD28 antibodies and injected into RAG1-deficient recipient mice (4 millions/mouse). Mice were treated or not with anti-NK1.1 antibody (200 µg per mouse; PK136, BioXcell) every 2-3 days. Spleens were harvested and analyzed by flow cytometry seven days after T cell injection for the presence of T cells. (n=4-5 mice/group). Significance calculated with two-tailed Mann-Whitney test.
